# Gender Differences in Oxidative Stress in Relation to Cancer Susceptibility and Survival

**DOI:** 10.3390/antiox12061255

**Published:** 2023-06-11

**Authors:** Alessandro Allegra, Santino Caserta, Sara Genovese, Giovanni Pioggia, Sebastiano Gangemi

**Affiliations:** 1 Division of Hematology, Department of Human Pathology in Adulthood and Childhood ‘Gaetano Barresi’, University of Messina, 98125 Messina, Italy; 132588@polime.it; 2 Institute for Biomedical Research and Innovation (IRIB), National Research Council of Italy (CNR), 98164 Messina, Italy; sara.genovese@cnr.it (S.G.); giovanni.pioggia@irib.cnr.it (G.P.); 3 Allergy and Clinical Immunology Unit, Department of Clinical and Experimental Medicine, University of Messina, 98100 Messina, Italy; gangemis@unime.it

**Keywords:** gender differences, cancer, oxidative stress, estrogens, testosterone, antioxidant, reactive oxygen species, sex hormones, mitochondria

## Abstract

Genetic, developmental, biochemical, and environmental variables interact intricately to produce sex differences. The significance of sex differences in cancer susceptibility is being clarified by numerous studies. Epidemiological research and cancer registries have revealed over the past few years that there are definite sex variations in cancer incidence, progression, and survival. However, oxidative stress and mitochondrial dysfunction also have a significant impact on the response to treatment of neoplastic diseases. Young women may be more protected from cancer than men because most of the proteins implicated in the regulation of redox state and mitochondrial function are under the control of sexual hormones. In this review, we describe how sexual hormones control the activity of antioxidant enzymes and mitochondria, as well as how they affect several neoplastic diseases. The molecular pathways that underlie the gender-related discrepancies in cancer that have been identified may be better understood, which may lead to more effective precision medicine and vital information on treatment options for both males and females with neoplastic illnesses.

## 1. Introduction

### 1.1. General Considerations on Gender Differences in Cancer Susceptibility

Epidemiological studies consistently demonstrate that there are gender variations in cancer incidence and mortality [1,2]. An analysis of the IARC’s global cancer statistics highlighted that men were more likely than women to develop cancer in 32 out of 35 tumor sites. The authors concluded that the causes of the significant gender differences are unknown; in fact, in 13 of these sites, the discrepancies could not be accounted for by known risk variables [3]. Another study found that the incidence of cancer in nonreproductive organs is 1.8 times higher in men than in women [4]. Animal studies have revealed gender disparities in cancer incidences, even in rodents that were not exposed to any harmful substances [5]. One study on rats found 68 “male-specific” and 19 “female-specific” carcinogens that caused cancer, though the exact causes of this difference were not clear [6].

A preliminary review of the literature on these gender-specific carcinogens revealed that oxidative stress might be a key mechanism, particularly for male-specific carcinogens. In fact, it has already been hypothesized that oxidative stress might affect a patient’s susceptibility to developing cancer from chemical carcinogens, which is supported by the literature on gender disparities [7].

### 1.2. Oxidative Stress and Cancer

**Figure 1:**
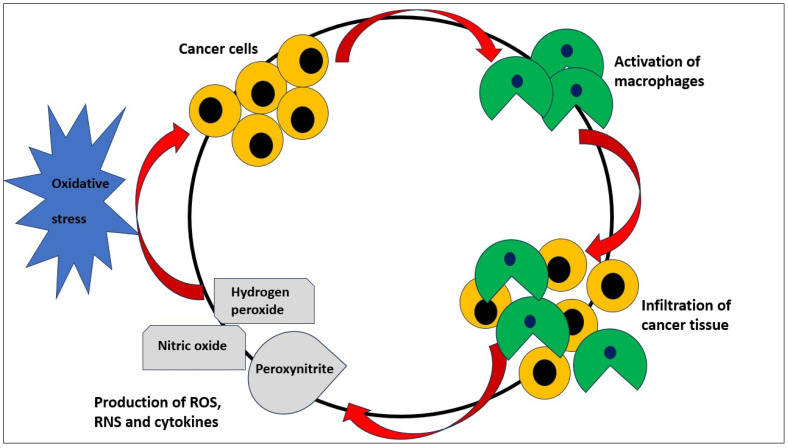
Cancer cells stimulate the activation of macrophages that infiltrate neoplastic tissue, with the consequent production of ROS, RNS, and cytokines production and, in turn, oxidative stress. Oxidative stress, as in a cycle, promotes cancerogenesis.

The imbalance between the production of toxic reactive species (TRS) and antioxidant defense mechanisms is referred to as “oxidative stress”. Reactive oxygen species (ROS) and reactive nitrogen species (RNS) are two categories of TRS. Superoxide anion, hydrogen peroxide, singlet oxygen, hydrogen superoxide, and reactive hydroxyl radical are the main components of ROS. The two RNS that are most known are nitric oxide and peroxynitrite [8]. Since ROS can damage DNA, proteins, and lipids within cells, oxidative stress is regarded as a serious condition. In cancer cells, TRS can come from a variety of sources. The chief contributors to the generation of ROS are thought to be an active metabolism and issues with the mitochondrial respiratory chain [9]. Additionally, activated macrophages infiltrate cancer tissue, increasing the inflammatory state and escalating the production of ROS and cytokines [10]. Moreover, the activation of oncogenes such as RAS2 or c-Myc is one example of a disorder in cellular signaling that is thought to be a significant generator of ROS [11,12]. For cells, oxidative stress may be damaging, but intrinsic oxidative stress in cancer cells in malignant neoplasms may have dramatic effects, including cancer cell proliferation, the promotion of genetic instability, and changes in cellular sensitivity to anticancer agents, and the modulation of cellular redox parameters is a real possibility [13,14,15,16,17,18] (Figure 1).

### 1.3. Gender Differences, Oxidative Stress, and Cancer

In one review, the literature on twenty-six recognized human carcinogens (IARC group 1) was examined. The analysis was based on about 600,000 abstracts and Tox21 screening assays and suggested a connection between testosterone, oxidative stress, and male-specific cancers. This would seem to indicate that the higher susceptibility to cancer seen in men may be due to a cellular response to oxidative stress that is only found in men [19].

Males are thought to experience oxidative stress more frequently than females [20], as confirmed in many other species, including flies, mice, and rats [21,22,23]. However, this hypothesis seems to depend on the cell type or tissue [22,23,24,25,26,27,28].

Other experimental data seem to confirm this hypothesis. NADPH oxidase activity and function appear to be lower in females, according to several studies [29]. Firstly, estrogen can directly cause reduced NADPH oxidase activity in females. Secondly, females have lower levels of p47, which is necessary for the assembly of the NADPH oxidase enzyme, as well as lower levels of superoxide production independently of estrogen. Females with lower levels of oxidative stress thus have lower levels of superoxide (Table 1).

In addition to gender differences in ROS generation, clinical and experimental studies have indicated that women have stronger antioxidant potential than men [30]. This may be because estrogen has antioxidant qualities, making women less vulnerable to oxidative stress [31]. Since postmenopausal women do not benefit from the anti-inflammatory and antioxidant protective properties of estrogen, they are more likely to experience increased oxidative stress [32]. Superoxide dismutase (SOD) transforms superoxide anions into hydrogen peroxide, and it seems that different tissues may react differently to this process.

Female rats apparently have higher heart SOD activity levels than male rats, and the levels of SOD activity in the brain and lungs seem to be higher in females [22]. Surprisingly, castration significantly reduced the levels of SOD activity in both male and female rats compared with the corresponding controls [22], thus indicating that sex hormones may be related to SOD activity levels. SOD activity was reported to be higher in female erythrocytes than male erythrocytes in one human experiment [33] and higher in female plasma than male plasma in another [33].

Glutathione peroxidase (GPx) is another enzyme that detoxifies hydrogen peroxide into water and oxygen. Progesterone and testosterone and other sex hormones control how active the GPx enzyme is. They are tissue-dependent in terms of sex-related differences, while male mice seem to display higher GPx activity than females in the heart, and in females GPx, activity is primarily in the kidney and brain [34]. Previous research has also revealed higher GPx activity in the brain and liver of female rats [35] and that the activity of GPx was more than twice as high in the hepatic mitochondria of female rats as compared with male rats of the same age [36]. In addition, female rats had more GPx activity in their livers than did male rats [37], and in humans, teenage girls had higher GPx activity in their blood than did men [38].

Since estrogen replacement therapy appears to increase erythrocyte GPx activity significantly in postmenopausal women and shows a positive correlation between GPx and serum estrogen levels in both premenopausal and postmenopausal women, it is likely that estrogen stimulates GPx expression. In fact, total hysterectomy in premenopausal females reduced the mRNA expression of SOD and GPx, which was then restored by estrogen replacement treatment, but had no effect on the expression of catalase [39].

Hepatic mitochondrial GSH levels in female rats are higher than in male rats, which appears to lead to a lower glutathione (GSH) concentration.

However, after ovariectomization, the levels of mitochondrial glutathione in the rats dropped to levels comparable to those in males.

Adolescent girls likewise had a greater blood GSH/oxidized glutathione (GSSG) ratio than did men [38], while men presented statistically significantly higher values of oxidized-reduced GSSG/GSH and GSSG in terms of concentration [40]. Additionally, premenopausal women that had had hysterectomies showed a decrease in GSH concentration and an increase in GSSG and GSSG/GSH ratio after 30 days. Interestingly, estrogen replacement therapy brought glutathione levels back to what they were prior to hysterectomy, highlighting once again the critical role of estrogen in the glutathione cycle [39].

Obesity is also a factor in the possible link between gender differences, oxidative stress, and the development of cancer. The burden of cancer attributable to obesity, expressed as population attributable fraction (PAF), is 11.9% in men and 13.1% in women for all obesity-related malignancies worldwide—though this clearly varies from region to region [41]. The highest PAF is typically seen in cases of esophageal adenocarcinoma in men and endometrial cancer in women.

The overall strong relationship between obesity and gynecological cancer (endometrial, postmenopausal breast, and ovarian cancers) suggests that female sex hormones have a role in the etiology of cancer. The risks for various cancers, including colon, rectal, gallbladder, kidney, and pancreatic cancers, are associated differently by gender depending on BMI and other somatometric factors [42,43]. This discovery emphasizes the harmful impact of visceral adiposity and insulin resistance in colon cancer, as well as the protective benefits of endogenous estrogenic effects against colon cancer in women [44,45,46]. Men are more likely than women to develop visceral adiposity.

Given that it is a chronic inflammatory state [47], obesity has been implicated in the initiation and progression of cancer [48]. This is due to the presence of numerous inflammatory components in the tumor microenvironment that support a malignant phenotype. Obese patients with metabolic abnormalities and adipose inflammation have a greater chance of developing cancer [49].

Tumor promotion is aided by ROS generation, which has also been linked to obesity [50]. The formation of ROS and the release of proinflammatory cytokines are induced by hyperglycemia combined with increased levels of free fatty acids, which together cause mitochondrial and DNA damage [51]. Furthermore, the folding of proteins is affected by oxidative stress. Obese people have higher amounts of free fatty acids (FFAs), which are linked to endoplasmic reticulum (ER) stress in adipocytes [52]. FFAs cause the production of ROS, which oxidize proteins and raise the proportion of unfolded proteins in ER. An inflammatory reaction is brought on by the build-up of unfolded proteins [53]. Cytokines have been connected to colon cancer in this build-up [54].

All these factors could help explain the different incidence of obesity-induced neoplasms in the two sexes.

**Figure 2:**
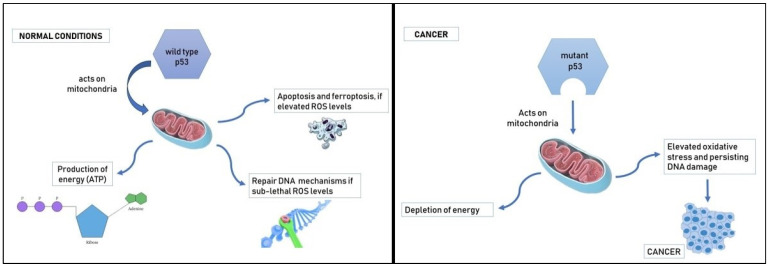
Mutant p53 is associated with a reduction in ATP production, elevated oxidative stress, and persisting DNA damage, which causes cancerogenesis.

Finally, the molecular processes underlying the links between gender disparities, oxidation, and the development of cancer have been defined in certain research. The main operating factor in the defense against cancer is the tumor suppressor protein p53, which plays a crucial role in protecting against long-term DNA damage. As a transcription factor, p53 promotes the expression of its target genes by interacting with DNA responsive sequences in their regulatory regions [55]. Through the p53-DREAM pathway, which includes its primary transcriptional target, the cyclin-dependent kinase inhibitor 1A, also known as P21, which is encoded in CDKN1A, can also suppress other sets of genes [56]. There is growing evidence that certain cancer sex disparities are related to differences in p53 functional abilities between males and females [57]. This suggests that either innate or externally imposed effects prevent p53 from conducting its functions equally between the sexes [58] (Figure 2). A change in p53 function results in a decrease in the quantity of mitochondria, and wild-type p53 has the ability to affect mitochondrial biogenesis. Additionally, p53 appears to be able to stop mitochondrial DNA mutation [59,60]. Interestingly, mitochondria may be more adapted to female circumstances than to male situations because they are exclusively of maternal origin [61]. As a result, an increase in ROS promotes the accumulation of p53, because p53 is tightly tied to redox processes [62]. The p53 activation pathways that result in ferroptosis, or programmed cell death, depend heavily on acute ROS stimulation [63]. Wild-type p53 can be made ready to activate repair pathways during a brief cell cycle arrest by sublethal ROS concentrations. However, DNA alteration, such as the TP53 mutation, puts the development of cancer at risk from sustained ROS levels at sublethal dosages [64]. Therefore, p53 mutation may play a role in the molecular pathways underlying sex differences in cancer risk.

### 1.4. Gender, Oxidative Stress, and Immunity

Innate and adaptive immune responses differ between males and females, as do their immunological responses. Immunological sex differences between men and women can be seen throughout their life but particularly after puberty and before reproductive senescence, which suggests that hormones may be at play in some cases. Females tend to have higher innate and adaptive (humoral and cellular) immune responses than males. Genetic and epigenetic factors, sex hormones, and a distinct response to inflammatory stimuli (e.g., oxidative stress) may be the cause of the higher immune response in females.

The leukocyte cell count in peripheral blood is similar in men and women [65]. Although monocyte and lymphocyte counts have not been found to differ by sex overall [65], gender dimorphism has been noted in several lymphocyte subsets and natural killer cells. As a result, men seem to have a greater percentage of CD3-CD56+ natural killer cells and CD8+ T-cytotoxic lymphocytes, whereas women exhibit a greater proportion of CD4 + T-helper cells, which leads to a higher CD4/CD8 ratio [66].

When discussing cytokine differences between the sexes, it is important to differentiate between cytokine levels in the absence of a challenge and cytokine production in response to various stimuli. Men seem to have higher amounts of proinflammatory cytokines in relation to basal inflammation [67,68].

Oxidative stress is known to activate and stimulate the NF-B transcription factor, which in turn causes the synthesis of a number of proinflammatory cytokines [69], and this might be because of their greater levels of oxidative stress. Females have a higher immunological and inflammatory response than males in terms of the sex differences in cytokine production in response to a stimulus [70]. As a result, specific immune cells in women respond to a challenge by producing more TNF and IFN than those in men [71,72].

Estrogen action has been linked to a greater synthesis of proinflammatory mediators in response to a challenge in females. As a result, normal estrogen concentrations increase the production of IL-6, IL-1, and TNF in response to the stimulation of human monocytes and murine macrophages [73]. On the other hand, removing endogenous estrogen tends to lower the proinflammatory response of the immune cells [74].

The above findings suggest that differences in the susceptibility and severity of malignancies could be caused by sex differences in immune system performance.

In the next section, we assess how the differences in gender and oxidative stress can affect the start and progression of neoplastic disorders.

## 2. Sex Differences in Oxidative Stress and Neoplastic Diseases

### 2.1. Glioma, Oxidative Stress, and Gender Differences

Although it is the second most common cancer in children, brain cancer is an uncommon condition in comparison with other cancer types [75]. Men are twice as likely to develop medulloblastoma, ependymoma, and gliomas than women, according to epidemiologic research [76]. In addition, a recent study found that women outlived males and responded better to standard treatment, identifying transcriptome signatures for glioblastomas in women [77].

Since oxidative stress and inflammation are also involved in the onset and progression of brain cancer, substances able to modify oxidative stress such as phytoestrogens have been considered good candidates for brain cancer prevention due to their antioxidant and anti-inflammatory properties. In fact, consuming foods containing phytoestrogens, particularly daidzein, appears to have a protective effect against gliomagenesis, according to an epidemiologic study conducted in 2006 [78]. Additionally, new research has shown that the phytoestrogens formononetin or biochanin A and the cytotoxic drug temozolomide combined have an enhanced anticancer effect in glioblastoma multiforme cells, with greater inhibition of cell signaling and invasion pathways and restoration of mitochondrial function [79,80].

Furthermore, long-term research has been conducted on the effects of gender on oxidative stress in the brain, including free radical generation, oxidative damage, and antioxidant enzyme levels and/or activity [81]. According to certain studies [82,83,84,85,86,87,88,89,90,91], male rats have greater DNA, protein, and lipid oxidative damage than female rats. The increased ROS generation in male rats [92,93] and the decreased levels and/or activities of antioxidant enzymes [94,95,96,97,98] are the causes of this oxidative damage. However, although these studies suggest that female rats have better redox homeostasis than male rats, other reports [99,100,101,102] have found no differences.

In terms of sex hormones, 17-estradiol (E2) and progesterone, which are produced by females, have neuroprotective effects in vivo and in vitro at physiological concentrations [103,104,105,106], but androgens and testosterone, which are produced by males, typically have neurotoxic effects [107]. The ability of some neurons and glial cells to create neurosteroids—sex hormones that are often produced de novo and independently of peripheral tissues—is particularly intriguing. These neurosteroids are equivalent to circulating steroids in both chemical and biological terms [108,109].

Along with oxidative stress, brain tumorigenesis has also been linked to decreased responses from nonenzyme (reduced glutathione, GSH) and enzyme antioxidant systems (SOD, catalase, and GPx) [110]. Since the central nervous system (CNS) is extremely susceptible to free radical damage, an imbalance between the production of free radicals and the effectiveness of the antioxidant defense systems is able to initiate the neoplastic process [111]. This theory is supported by numerous research works. For instance, research has shown that subcutaneous administration of hydroxytyrosol, but not oleuropein or a combination of both compounds, resulted in a significant inhibition of tumor growth through mechanisms involving endogenous enzymatic and nonenzymatic antioxidant defense systems [112,113,114].

Thus, the existence of gender differences in processes related to brain tumors, such as the management of redox status, suggested that research on brain cancer should take gender differences into account in preclinical studies, screening, and prevention programs, as well as in therapeutic approaches.

### 2.2. Liver Cancer, Oxidative Stress, and Gender Differences

Liver cancer is currently the second most common cancer type [115]. The 5-year survival rate for people with liver cancer only oscillates by 10%, despite the use of intensive treatments [116]. In total, 90% of liver cancer cases are caused by hepatocellular carcinoma (HCC).

Even after accounting for variations in exposure to risk factors, there is a two- to four-fold higher incidence of liver cancer in men than in women in humans [117,118]. Additionally, males predominate in transgenic mouse models of hepatitis virus infection and models of liver tumor induction in mice after exposure to chemical carcinogens such as AFB1, 4-aminobiphenyl (ABP), and diethylnitrosamine (DEN) [119]. Additionally, numerous human and animal studies on HCC confirmed sexual dimorphism during the onset and development of alcohol liver disease (ALD). It is likely that variations in the expression of genes that code for ethanol-metabolizing enzymes have an impact on the development and progression of ALD and liver cancer [120]. Alcohol dehydrogenase (ADH) activity varies between sexes; it is lower in men than in women, which leads to less acetaldehyde build-up. Additionally, studies reveal that estrogens positively affect CYP2E1 and ADH, indicating that ethanol should be metabolized more quickly in females than in males [121].

Some studies demonstrated that male mice are more vulnerable than female mice to HCC [122].

It should be mentioned that lipid peroxide levels in the liver and serum are decreased by estradiol and its derivatives, which are potent endogenous antioxidants [123,124]. The loss of SOD and glutathione peroxidase activity, as well as iron (ferric nitrilotriacetate)-induced ROS production, lipid peroxidation, activation of AP-1 and NF-B, are all suppressed by estradiol in cultured rat hepatocytes, according to recent research [125,126]. In isolated rat liver mitochondria, estradiol also reduces the lipid peroxidation brought on by iron [125]. These results imply that the inhibitory impact of estradiol on AP-1 and NF-B activation may result from scavenging ROS and/or from lowering intracellular ROS generation by inducing antioxidant enzymes.

Male sex, like the viral risk factor for hepatic fibrosis, is a significant risk factor for HCC [127], while it is unknown whether males and females differ in their susceptibility to the integration of viral DNA, which causes the malignant transformation of hepatocytes. In contrast, premenopausal women are least susceptible to HCC because they lack the risk factors of older age and male sex. In a study, 901 individuals with HBV-associated HCC had their age-specific male-to-female ratios looked at. The younger group had a smaller percentage of females (10.5%) than the older group when the subjects were split into two age groups based on whether they were younger or older than the menopausal age of 50 years.

The differences in hepatic damage were connected to alterations in cellular GSH, ROS production, and cell REDOX status brought on by the metabolism of ethanol. The imbalance between acetaldehyde and ALDH is accentuated by CYP2E1 induction, which also leads to the production of ROS, the subsequent depletion of GSH, and oxidative damage [122]. Similar results were obtained employing a different experimental model exposing mice to aminobiphenyl (ABP) [128,129,130,131].

In contrast, levels of the hepatotoxicity biomarker alanine aminotransferase (ALT) were acutely two-fold higher in male adult mice exposed to ABP, DEN, or carbon tetrachloride (CCl4) than in female adult mice [132], while levels of the inflammatory biomarker interleukin-6 (IL-6) did not differ based on sex. While CCl4 produced a 40-fold ALT elevation but without sex differences, treatment of immature mice with either ABP or DEN using conventional tumor-inducing postnatal exposure protocols did not result in an increase in serum ALT or IL-6 levels in either males or females. There was no sex difference in the baseline expression of Ggt1 or Hmox1, but adult females expressed the NRF2-responsive gene Nqo1 at higher levels than adult males. Animals that were still developing sexually revealed no sex difference in the three genes’ baseline expression. While CCl4 slightly increased the expression of Ggt1 in both males and females and Nqo1 only in females, postnatal DEN exposure slightly increased the expression of Ggt1 only in male mice and Nqo1 in both sexes. Together, these findings rule out the possibility that postnatal carcinogen exposure in mice results in acute hepatotoxic, inflammatory, or NRF2-activated gene responses that are responsible for the male predominance in liver tumor growth [132]. These results also imply that when extrapolating putative processes to liver carcinogenesis models that frequently employ postnatally exposed mice, acute toxicity studies conducted in adult mice should be read with caution. However, the various experimental setups used could be the cause of the disparate results found in the various studies.

Aflatoxin B1 (AFB1) is a strong hepatotoxin and hepatocarcinogen for humans and most other mammalian species, although adult mice are remarkably resistant to it [133]. *Aspergillus flavus*, a mold that develops on groundnuts, grain, and maize that mice frequently consume, produces AFB1. Cytochrome P450 (CYP) transforms AFB1 in both humans and mice into a reactive AFB1-epoxide that can damage DNA by attaching to the N-7 atom of guanine [134]. Once produced, the glutathione S-transferase (GST) enzymes in the cytosol can catalyze the conjugation of the AFB1-epoxide with reduced glutathione to detoxify it. Water-soluble aflatoxin mercapturic acids (AFB1-NAC) are eliminated in urine as glutathione conjugates of AFB1-epoxides [135]. Mice’s inherent resistance to AFB1 may be due to CYP isoenzymes’ poor capacity to produce reactive epoxides and/or GST isoenzymes’ great capacity to produce glutathione conjugates.

The important function of GSTA3 in AFB1 resistance was confirmed by a study that produced glutathione S-transferase (GST) A3 knockout (KO) mice. GSTA3 KO mice are vulnerable to the acute cytotoxic and genotoxic effects of AFB1 [136]. In contrast to the known higher incidence of liver cancer in males in humans, this study shows that initial vulnerability to AFB1 is greater in female mice and that oval cell response and GSTA3 peroxidase activity may affect susceptibility to cancer development (Table 2).

Other information supports the notion that oxidative stress plays a part in the different onset of liver cancer in the two sexes. According to a study, age-related TBARS accumulation in the liver may be sex-related, because it was more noticeable in old male mice compared with old female mice. Gonadotropic hormones, particularly estrogens, may be the cause of these sex-related variations in the TBARS level [137]. The connection between estrogens and liver oxidative damage has been shown by numerous in vitro investigations [138]. Since females at that age are in a reproductive decline stage, hormonal changes alone cannot account for the fact that TBARS in 18-month-old females were higher than in males of the same age. The growth of tumors seen in aged male mice may be linked to gender-specific changes in TBARS. These findings are consistent with some published studies that link declining lipid peroxidation (LPO) levels to increasing tumor size [139].

Researchers have studied the activities of total superoxide dismutase (tSOD), Gpx, and catalase (CAT). LPO, quantified in terms of TBARS, was determined by the authors to be a marker of liver oxidative damage. LPO increased with aging in both sexes. In both mouse sexes, tSOD appears to be a dormant antioxidative enzyme. The principal alterations in the liver’s antioxidant capacity of aging mice were connected to sex-related increases in CAT and Gpx that were only seen in males. Surprisingly, hepatic tumors developed in more than 60% of 18-month-old men (but not girls), which first appeared at 10 months. The findings indicate that increased liver antioxidant capacity of CAT and Gpx in male mice may be an indication of oxidative stress; increases in CAT and Gpx activities in male mice are strongly correlated with the incidence of hepatic tumors; and significantly increased SOD activity in tumor-bearing mice may have been caused by damage from accumulated hydrogen peroxide H_2_O_2_ [140].

The varied ways that oxidative stress behaves in the two sexes is also intriguing. An experiment revealed that during male senescence, CAT and Gpx significantly changed. In contrast to this, there was little to no change in CAT activity and no appreciable change in Gpx activity in female mice. In general, CAT and Gpx activity were 50% and 85% higher in males than in females. Tumor-bearing mice displayed elevated tSOD activity in contrast to the antioxidant enzyme status of tumor-free mice (inert tSOD activity). Antioxidant enzyme activities are typically thought to vary during or after tumor development [141]. Most past investigations have suggested that cancer has poor antioxidant enzyme activity [142]. However, most of them used cell lines, and in some of them, conclusions were reached based on blood sample activity measurements that did not accurately reflect the enzyme levels in the tumor or the affected organ. Manganese superoxide dismutase (MnSOD) expression has been shown to be high in many human cancers and, in some tumors, the level of MnSOD is directly correlated with the tumor grade [143]. Additionally, Manna et al. demonstrated that MnSOD overexpression in tumors may give tumor cells a survival advantage [144]. Another author’s theory is that tumor cells produce a significant amount of H_2_O_2_ [145], and research showing that tSOD overexpression promotes H_2_O_2_ generation supports this idea. To fulfil the demands of the increased LPO and H_2_O_2_ build-up brought on by the increased SOD activity, these facts may explain why males generally have higher CAT and Gpx activities [146].

Numerous studies have demonstrated that oxidative stress restricts the ability of cells to undergo mitosis, suggesting that oxidative stress may also condition a different proliferative capacity of cancerous cells [147]. Based on higher antioxidant enzyme levels and the oxidative stress situation prevalent in men, it is possible to infer that cell division favoring clonal growth can occur. Such a phenomenon might aid in the development of cancer. Similar findings have been published from Gonzales, where higher antioxidant levels have been linked to a faster rate of cell division [148]. Like the gender difference in the incidence of liver cancer in humans, postnatal exposure of mice to ABP causes a higher incidence of liver tumors in males than in females. ABP-DNA adducts that start tumor growth are produced because of first N-hydroxylation that is initially mediated by CYP1A2, according to a conventional theory of ABP carcinogenesis. CYP2E1 was found to be a key ABP N-hydroxylating enzyme in isozyme-selective inhibition tests employing liver microsomes from wild-type and genetically engineered mice. Oxidative stress was brought on by the N-hydroxylation of ABP by transiently expressed CYP2E1 in cultured mouse hepatoma cells. Male wild-type mice exposed postnatally to a tumor-causing dosage of ABP also experienced oxidative stress, but neither male Cyp2e1(/) mice nor female mice did. However, females showed a stronger NRF2-associated antioxidant response [149]. These results imply that CYP2E1 is a novel ABP-N-oxidizing enzyme and that sex differences in tumor incidence and cell proliferation may be related to sex differences in oxidative stress and antioxidant responses to ABP.

Finally, a particularly exciting area of research focuses on the relationships between gender differences, obesity, oxidative stress, and liver cancers. Recent population-based studies have repeatedly demonstrated that obese men are far more likely to acquire HCC. Men with a BMI of 35 kg/m^2^ showed a severe 4.52-fold increase in relative risk of mortality from liver cancer, although women only showed a small 1.68-fold increase, according to prospective research involving more than 900,000 persons [150]. The large gender-based variation in HCC incidence has been further validated by a cohort study of 5.24 million persons in the UK [151]. According to the studies, BMI and HCC in males were correlated [151], and increased and disordered ROS production in extra adipose tissue during obesity may increase oxidative stress and the likelihood of developing HCC [152]. In contrast to subcutaneous fat accumulation, visceral fat deposition is substantially higher in males than in females [153]. In numerous datasets [154,155], men were found to have larger visceral fat and liver fat contents than women, despite having similar total fat and BMI values. Liver cancer is facilitated by visceral fat, which actively secretes carcinogenic adipokines that cause persistent inflammation. High androgen receptor density may be the root cause of the differences between liver cancer and visceral fat accumulation [156]. As people get older, their visceral body fat increases, while their subcutaneous body fat decreases, which is correlated with an increase in the incidence of HCC [157].

### 2.3. Colorectal Cancer, Oxidative Stress, and Gender Differences

Colorectal cancer (CRC) is the second most prevalent cause of cancer mortality among men and women globally [158]. Drug resistance and adverse reactions continue to hinder the success of treatment, despite the fact that the overall survival rate of CRC patients has increased because of advancements in treatment methods such as chemotherapy.

According to certain research, the disease affects people of various sexes at different rates, and this could be due to oxidative stress. For instance, neutrophils and monocytes both contain the lysosomal enzyme myeloperoxidase (MPO) [159]. Hypochlorous acid, a potent oxidant produced by MPO for its microbicidal function, can target proteins, nucleic acids, and unsaturated lipids by simultaneously releasing ROS [160]. A-463 G>A transition, which is situated in the consensus binding location of the SP1 transcription factor, is a frequently occurring polymorphism in the MPO gene promoter region. In vitro, the MPO G wild-type allele confers approximately twenty-five times more transcriptional activation than the -463 A variant. According to reports, this polymorphism raises the likelihood of developing laryngeal, lung, breast, and stomach cancers [161,162,163,164,165]. According to a study, those with the genotype GA/AA were considerably less likely to get colorectal cancer than people with the GG genotype. The reduced risk was particularly significant among men according to the stratified analysis. For male individuals with the GA/AA genotype compared with GG genotype, the adjusted OR was 0.47. However, among women, the OR was not statistically significant. The possibility that estrogen-induced increased MPO-463 A promoter activity is the cause of the MPO-463 A variant’s lack of protective effect in female patients is therefore plausible [166].

Oxidative stress and cancer have been linked in other research. Bilirubin is more than only the byproduct of heme catabolism. It is now thought to be an essential blood component that forms endogenously and has anti-inflammatory and antioxidant activities [167,168,169,170,171,172]. Recent research has indicated that bilirubin, particularly unconjugated bilirubin (UCB), may provide protection against oxidative stress-related illnesses such as CRC. In vitro research outcomes also demonstrated that UCB has antimutagenic qualities [173], which may be especially pertinent for gut health. Tetrapyrroles, a family of bile pigments that are abundant in the intestine, reduced the genotoxicity brought on by poly-/heterocyclic amines and triggered apoptosis in cancer cells [174,175,176]. Higher circulating UCB concentrations were positively linked with CRC risk in males and negatively associated with risk in women, according to a study that examined relationships between UCB and CRC risk in the European Prospective Investigation into Cancer and Nutrition (EPIC) study [177]. According to one study, every one standard deviation increase in log-UCB was associated with a lower risk of CRC in males and a higher risk in women (heterogeneity = 0.4 for differences between men and women) [178]. Finally, it has been demonstrated that UCB may easily cross cell membranes in vivo, infiltrate colon cancer cells to stop tumor cell growth [179], trigger death in cancer cells in vitro [180], and control gene transcription (via ERK, p53, and p27) [181]. Strogen, lower NADPH-oxidase activity, or other previously described mechanisms may make women less susceptible to oxidative stress [23].

### 2.4. Lung Cancer, Oxidative Stress, and Gender Differences

Lung cancer is the most common cancer in the world [182,183]. There may be gender disparities in lung cancer incidence, according to epidemiologic data [184,185,186]. Agreeing to several studies, women may be more likely than men to acquire lung and colon cancer from smoking cigarettes [187,188].

The expression of genes relevant to cancer and the immune system is altered by genetic and epigenetic alterations, as well as by the abnormal expression of noncoding RNAs, which predisposes the lung epithelium to carcinogenesis. Smoking-related oxidative stress contributes to decreased genomic integrity and promotes epithelial–mesenchymal transition and the creation of a chronic inflammatory milieu. Although not all smokers develop lung cancer, this results in abnormal immune reactions that support the development of cancer. Females are more likely to accumulate oxidative stress damage due to gender differences in the metabolism of cigarette smoke, which increases their risk of developing lung cancer [189]. Additionally, ROS and RNS can activate signaling molecules such as HIF1, which is a key regulator of angiogenesis and a driving force behind the development of tumors [190]. Furthermore, it has been demonstrated that the byproducts of ROS and inflammation can inactivate PTEN, a tumor suppressor gene that is frequently altered in lung cancer, by creating an intramolecular disulfide bond [191,192].

Large epidemiological studies have demonstrated that for every pack-year of smoking, women are two to three times more likely to die from COPD than males [193] and are 50% more likely to develop COPD than men. One explanation is that because women’s lungs are smaller than men’s with comparable smoking histories, the harm from oxidative stress is more obvious in women [193]. Another is sex variations in the metabolism of tobacco: women have higher liver CYP1A1 and CYP1B1 activity levels, which activate specific tobacco smoke components to create ROS [194]. Strogen’s role in activating CYP enzyme-related pathways is a contributing factor in the enhanced CYP expression in females [195]. For instance, a study of smokers who developed lung cancer showed that females had higher levels of CYP1A1 expression and a commensurate rise in DNA adducts, even in lung tissue that was not cancerous [196]. Additionally, studies on animals showed that the injection of naphthalene—a substance found in tobacco smoke—caused more airway damage in female mice than in male mice. This was due to increased CYP enzyme expression and the production of metabolites, which led to a more severe inflammatory response in the airways and produced more ROS than in male mice [197]. Because women are more frequently exposed to biomass smoke, exposure to indoor and outdoor air pollution is also a significant risk factor for the development of lung cancer in nonsmokers [198,199].

The varied ways that oxidative stress affects the incidence of pulmonary neoplasia in the two sexes could be explained by other processes, as reported in studies performed employing a class of pervasive environmental pollutants known as polycyclic aromatic hydrocarbons (PAHs) [200,201,202].

A study identified sixteen environmental PAHs in workplaces and assessed that women who worked in the office, next to the coke oven, or on its bottom or side, respectively, had significantly higher urine 8-OHdG and 8-isoPGF2a levels and lymphocytic micronucleus frequencies than men who worked in those locations. Gender and BPDE-Alb adducts had a strong impact on rising micronucleus frequencies. The foregoing gender disparities were more pronounced in the median- and high-exposure groups, according to authors who further stratified all workers based on the tertiles of urinary ROH-PAHs or plasma BPDE-Alb adducts [203]. As a result, women were more vulnerable than males to the oxidative stress and chromosomal damage caused by PAHs, which could be additional evidence for gender differences in PAH-exposure-related lung carcinogenesis (Table 3).

### 2.5. Melanoma, Oxidative Stress, and Gender Differences

Since the middle of the 1950s, malignant melanoma prognoses for cases with advanced metastases have remained dismal [204,205]. Gender has been shown to be an independent prognostic factor of melanoma survival in numerous studies, as it remains significant after adjusting for nearly all known prognostic indicators, including age, Breslow thickness, Clark level of invasion, body site, histological subtype, and recently, emerged prognostic indicators, such as ulceration, sentinel node status, and mitotic rate [206,207]. Both the incidence and survival of malignant melanoma differ significantly across gender. Male patients advance more quickly to stage III [208] and maybe even stage IV melanoma [209,210]; male original melanomas appear to grow more quickly than those in females; and men present with nodal and visceral metastases more frequently than women [206]. Instead, women are more likely to present with tumors that are in an earlier stage, have longer survival times, and experience better outcomes [211,212,213,214,215].

More and more evidence points to the involvement of oxidative stress, which is brought on by high amounts of ROS, such as superoxide anions and hydrogen peroxide, in the development of melanoma [216,217]. When compared with nearby tissues or melanocytes, melanoma cells produce a lot of ROS, which they then excrete into extracellular space [218]. Additionally, melanoma cells have elevated intracellular ROS levels [219].

High amounts of oxidative stress are known to exist in the initial melanoma tumor environment [220,221,222]; tumor-related immune cells release ROS [223], and ultraviolet (UV) radiation further intensifies oxidative stress in the skin and melanocytes [224]. In contrast to surrounding nontumor tissue, benign melanocytic nevi, and control subject skin, Sander et al. discovered a considerable upregulation in antioxidant enzymes in human melanoma biopsies, indicating that the melanoma cells were responding to increasing oxidative stress [221].

According to a different study, the advantage that females have in terms of melanoma survival is likely due to sex differences in the capacity to counteract the oxidative stress brought on by ROS [220]. In fact, it appears that the oxidative environment in the skin of male and female mice has different baseline characteristics; UV-induced oxidative stress amplifies these differences. In comparison with female hairless mice, the skin of males had a lower baseline level of antioxidant enzymes and a roughly 10-fold lower antioxidant functional capacity. In comparison with levels found in the skin of male mice exposed to UVB radiation, the skin of female mice showed a significantly higher induction in antioxidant level, greater antioxidant functional capacity, and lower levels of 8-oxo-deoxyguanosine, the most common type of DNA damage caused by ROS [225]. These findings were supported by an experiment that looked at gender differences in the development of cancer linked to UV-induced chronic inflammation [226]. According to the finding’s, photoaging damage was present in both male and female mice at the ninth week. However, only male mice in the third week developed skin tumors. Additionally, UV increased the expression of the p65, p-p65, IL-6, and TNF proteins in skin, and these factors were more elevated in the male mouse model. The parameters of blood systemic inflammation were altered to variable degrees in the model groups, according to hematology data, whereas the internal organs of both model groups revealed varying degrees of inflammatory cell infiltration, according to pathology results. These findings suggest that UV-induced skin inflammation, carcinogenesis, and systemic damage differ between the sexes.

Additionally, it is possible that men’s higher ROS levels encourage the selection of ROS-resistant melanoma cells. Consequently, ROS can promote melanoma cells’ capacity for metastatic spread. Additionally, because men have weaker antioxidant defenses, the ROS that melanoma cells produce damage surrounding healthy tissues more severely, which promotes metastasis. As a result, ROS could account for the reported disparities in melanoma survival between males and females [220].

After menopause, according to some researchers, the female advantage vanishes [207]. Others, however, discovered that females continue to live longer even after menopause [227]. In female rats, ovariectomies boosted peroxide generation in liver cells to levels seen in male cells, decreased antioxidant enzyme levels to those found in male cells, and restored both peroxide and antioxidant enzyme levels in female cells to the control female levels [23]. This team discovered that 17-b-estradiol decreased hydrogen peroxide production when isolated mitochondrion was incubated with it [228].

The effect of antioxidant supplementation on the incidence of melanoma has also been studied; however, due to the small number of events in the trials, no significant effect [229], or even a negative effect [230], was discovered. More importantly, the effect of antioxidants varied by gender in each of these studies, affecting both the incidence of melanoma [230] and all cancers [231]. This strongly implies that gender has a role in the relationship between melanoma and ROS.

### 2.6. Non-Hodgkin Lymphoma, Oxidative Stress, and Gender Differences

The Swedish Lymphoma Register was used in a population-based cohort study that looked at gender differences in the incidence of lymphoma subtypes and excess mortality among people diagnosed between 2000 and 2019 [232]. Poisson regression was used to predict the male-to-female incidence rate ratios (IRRs) and excess mortality ratios (EMRs) after adjusting for age. They discovered 36,795 instances of lymphoma, 20,738 (56.4%) of which were in men and 16,057 (43.6%) in women. Incidence rate ratios (IRRs) ranged from 1.15 in follicular lymphoma to 5.95 in hairy cell leukemia, with men being considerably more at risk for 14 of the 16 subtypes of lymphoma. Although only statistically significant for classical Hodgkin lymphoma 1.26, aggressive lymphoma not otherwise specified 1.29, and small lymphocytic lymphoma 1.52, EMRs > 1 was seen in 13 out of 16 lymphoma subtypes, indicating higher mortality in men. Similar findings were obtained from a related analysis utilizing information from the Danish Lymphoma Register [232]. In conclusion, researchers found that for the majority of lymphoma subtypes, men had a significantly greater incidence and a tendency toward higher death rates.

The differing levels of oxidative stress experienced by the two sexes may contribute to the development and spread of lymphomas. For instance, a study in [233] examined the idea that lymphomagenesis following low-dose radiation is aided by mitochondrial malfunction and elevated superoxide levels in thymocytes overexpressing Bax (Lck-Bax1 and Lck-Bax38&1). Single whole-body doses of 10 or 100 cGy of 137Cs, iron ions, or silicon ions, were administered to Lck-Bax1 single-transgenic and Lck-Bax38&1 double-transgenic mice. In female Lck-Bax1 mice, a 10 cGy dosage of 137Cs markedly increased the incidence and development of thymic lymphomas. In contrast to silicon ions, a 100 cGy dosage of high-LET iron ions significantly and dose-dependently accelerated lymphomagenesis in both male and female Lck-Bax38&1 mice. Lck-Bax38&1 overexpressing animals were bred with Sirtuin 3 knockouts, a mitochondrial protein deacetylase that controls superoxide metabolism, to ascertain the contribution of mitochondrial oxidative metabolism. Significant increases in thymocyte superoxide levels and accelerated lymphomagenesis were seen in Sirt3//Lck-Bax38&1 animals [233] (Table 4). These findings demonstrate that radiation exposure increases lymphomagenesis in Bax overexpressing animals in a manner that depends on both LET and gender. These results are consistent with the hypothesis that in Lck-Bax transgenic mice, mitochondrial dysfunction increases superoxide levels and speeds up lymphomagenesis.

## 3. Gender Differences, Oxidative Stress, and Responsiveness to Anticancer Therapy

The possibility that oxidative stress in relation to gender may affect the effectiveness and toxicity of chemotherapy and radiotherapy in cancer subjects is unquestionably an exciting area for future research.

By interfering with mitochondrial function, bioenergetics, signaling pathways, and redox balance, anthracyclines can cause cell malfunction and death [234]. The majority of these targets exhibit sexual dimorphism, including “redox features” of cells, such as altered redox-associated molecules and enzymes in relation to gender differences in terms of intracellular production and biochemical activity of reactive species, as well as expression of genes related to mitochondria [235,236]. Along with pharmacodynamics, sex-related variations in pharmacokinetics may have significant clinical ramifications, since they impact the side effects of certain drugs.

Men appear to have a considerably higher doxorubicin clearance than women [237]. In fact, doxorubicinol levels have been found to be higher in men, which may be connected to greater aldoketoreductase activity [238]. A lower expression of p-glycoprotein in females may also cause doxorubicin and doxorubicinol to accumulate, increasing the risk of cardiotoxicity. The pharmacokinetics of epirubicin have also been shown to differ according to sex [238].

Anthracyclines’ overproduction of ROS and RNS causes redox stress, which then causes cardiac injury [239], DNA damage, lipid peroxidation, membrane injury, and/or apoptosis, as well as changes in the enzymatic activity of the mitochondrial redox system. The respiratory complexes, Krebs cycle enzymes, oxidative phosphorylation, oxidation, and nitric oxide synthases (NOSs) are among the enzymes changed [240,241,242].

The various oxidative stresses that influence cardiotoxicity in a gender-specific way could in theory be sustained by a complicated inter-relationship between estrogen receptors and enzyme activity engaged in redox processes. Adult male SH rats with tumors seem to be more cardiosensitive to doxorubicin treatment than female rats or hormone-deficient rats, which lends credence to this theory [243]. According to these findings, doxorubicin-induced cardiotoxicity is regulated by reproductive hormones, and the selective cytotoxic mechanism probably works by increasing oxidative stress and apoptosis in male SH rats [243].

The anthracycline drug doxorubicin (Dox) is highly effective against a number of neoplastic illnesses but also causes dose-limiting cardiotoxicity [244,245]. Congestive heart failure, for instance, occurs following Dox treatment at a rate of about 4% at doses of 500–550 mg/m^2^, but this rate rises to 18% at doses of 55–600 mg/m^2^, and it reaches 36% in patients receiving >601 mg/m^2^ of Dox [246]. A continued follow-up of the cardiac condition of patients that received anthracyclines is recommended, since cumulative and late-onset progressive cardiotoxicity might be seen even decades after treatment [247].

In the mitochondria, doxorubicin builds up and causes an excessive amount of ROS production. Male adults (15–55 years old) are more likely than females to have cardiovascular disease overall [248,249,250,251,252]. Doxorubicin treatment for women causes cardiac failure in 6–20% of adults and 40% of pediatric patients [13]. Additionally, postmenopausal women are more susceptible to cardiac stress than men their own age following Dox treatment [253]. Finally, Dox-induced cardiotoxicity in prepubescent girls was found to be more severe than in boys of the same age [253]. Additionally, according to recent clinical reports, male adults and young girls are more cardiosensitive to Dox. Adult male SHRs with tumors are more cardiosensitive to Dox than female or hormone-deficient animals. In fact, the selective cytotoxic mechanism is thought to work because oxidative stress and apoptosis are more strongly activated in male SHRs, and this suggests that Dox-induced cardiotoxicity inhibits or negatively regulates reproductive hormones [254].

Estrogen may act as a cardioprotectant by reducing left ventricular hypertrophy, preventing cardiomyocyte death, and protecting against the onset of cardiac fibrosis in females [255]. Estrogen, and possibly testosterone, may protect the heart against excessive drug-induced oxidative damage.

Although gender is known to affect how the body reacts to radiation, the underlying molecular mechanisms are not clear, and consequently, current risk estimates are uncertain and have low-resolution dose limits [256]. It is recommended to use male and female reference phantoms for study; however, few health authorities have defined dose limits with sex-specific regimens [257].

Predicting cellular outcomes from proteins related to DNA damage and repair has highlighted the qualitative variations in ionic radiation (IR)-induced reactions between age and sex. Juvenile girls and males appear to start separate signaling cascades as opposed to merely altering the response intensity of the same mechanism. Although both share a suppression of cell cycle progression, males appear to shift towards proapoptosis with mitochondrial stress and reduced DNA repair, while females display activated DNA repair and prosurvival mechanisms. Inflammatory regulators also seem to compete for control over the activation and inhibition of immunological responses; however, they were not found in females [258].

To comprehend the effects of ionizing radiation, radiobiology is key. The interaction of radiation with targeted and nontargeted cells, tissues, and organs has profound implications on both the early and late development of primary and secondary malignancies, but knowledge of the mechanisms underlying radiation carcinogenesis is still lacking. Some studies, however, have suggested that nontargeted effects may contribute to a higher risk of developing cancer [259].

Males and females experience different rates of radiation-induced mutation patterns, subsequent changes in gene expression and epigenetic status, and malignancies [260,261,262,263]. According to research by Korturbash et al., local cerebral irradiation of mice causes DNA damage and changes in global DNA methylation that are tissue- and sex-dependent. They demonstrated that although nontargeted effects can result in skin hypomethylation, they have not been studied for spleen hypomethylation. They also found that males appear to be more obviously hypomethylated than females [264]. In nontargeted tissues, similar outcomes have been seen for the control of the microRNAome and inflammatory responses [265,266].

Following exposure to X or gamma rays, oxidative DNA damage and cell death are brought on by free radical production which is due to the interaction of ionizing radiation with water molecules and redox-mediated biological pathways. In both the nucleus and the mitochondria, the interaction of free radicals with DNA causes various forms of DNA oxidation. Further DNA damage can result from oxidative stress, inflammatory reactions, and cell death due to necrosis or apoptosis. Patients with cancer and those receiving radiation for their malignancies both have higher levels of oxidized cell-free DNA [267,268,269].

Precision radiation oncology in cancer treatment and individualized risk evaluation for ionizing radiation (IR) exposure are still in their infancy [270]. Improved medicines with fewer toxicities might be developed if there was a better understanding of the sex-related mechanisms of protection and harm [271].

Another approach would be to use natural substances for the modification of oxidative stress [272,273]. As mentioned above, several natural products have an enhanced anticancer effect via a restoration of mitochondrial function [79,80]. These substances could reduce the negative effects of sex on the progression of neoplastic diseases, perhaps allowing a reduction in the dosage of traditional anticancer drugs.

## 4. Final Remarks and Conclusions

A vast range of systems involved in the redox characteristics of cells are affected by oxidative damage [235,274]. Women often live longer than men as a result of the benefits of their X chromosomes, the antioxidant protective properties of estrogen, and a lower exposure to extrinsic risk factors such as alcohol and smoking. Sex hormones alter the expression of several crucial transcription factors that control ROS-induced stress and in vivo responses. Due to estrogen, women have lower levels of ROS generation and mitochondrial damage than men, which is also linked to increased mitochondrial function and disease resistance. Furthermore, estrogen benefits females by influencing NRF 2 activation and the regulation of other antioxidant-related transcription factors through NRF2. Effective cancer treatment necessitates an awareness of the potential of ROS and a focus on the traits of the study target, such as the patient’s gender [275]. ROS have a variety of biochemical targets in cells.

Males exhibit higher rates of oxidative damage than females [276,277,278]; in fact, after adjusting for smoking and body mass index, healthy males have a 29% higher level of urinary oxidative damage [279]. Males also express lower levels of antioxidants than females, such as GSH, catalase, and SOD. Given that oxidative damage can result in cancer and cardiovascular disease, this could explain why women generally live longer than men [280].

The genetic overexpression of antioxidant enzymes in females, which may be brought on by estrogen receptor activation, may also be important. In fact, ovariectomy-induced menopause in mice likely increases oxidative damage susceptibility. Although androgens, such as testosterone, appear to weaken these same defense mechanisms, estrogen levels do not entirely account for these variations in antioxidant defense [281].

Regarding mitochondrial function, the traditional view of mitochondrial inheritance holds that mtDNA is only passed down through the female line, yet some reports have suggested that it may also be inherited from the father [282,283,284]. The different male/female sex hormones that control mitochondrial energy, OXPHOS, and Ca^2+^ homeostasis, may be one of the causes of mitochondrial sexual dimorphisms [285]. Although this varies depending on the tissue in question and the age/hormonal status of the tested subject, female mitochondria typically have a greater functional capacity than male mitochondria [286].

The relationship between mitochondrial activity, gender variations, and cancers can thus be explained in a number of ways.

The thymidine phosphorylase (TP) enzyme contributes to the lowering of TP activity during the metabolism of pyrimidines. It disrupts the nucleotide pool by creating a build-up of thymidine and deoxyuridine. The result is aberrant mitochondrial DNA that displays point mutations, many deletions, and depletion [287].

In response to cellular stress conditions, such as inflammation and oxidative damage, TP overexpression takes place and stimulates the Pi3 kinase/Akt pathway, the apoptotic caspase 3/9 pathway, and the autophagic BNIP3 gene with an antiapoptotic activity that promotes proliferation [288,289]. TP is pathologically overexpressed in a number of human tumors and is associated with a bad prognosis [290]. The plasma of neoplastic patients has also been found to contain TP-related proteins [291].

TP overexpression is associated with the carcinogenesis process and may play a predictive function in breast cancer [292]. During the female menstrual cycle, the glandular and stromal epithelium contain the enzyme TP [293]. According to one study, cytoplasmic TP overexpression is associated with microvascular density in canine mammary tumors of a severe grade and may be an indicator of breast cancer [294].

The actions of hormones on ion channels also form a specific method by which sex hormones, particularly estrogens, influence oncogenesis. The growth and multiplication of cells depend greatly on potassium channels. The cell cycle’s G1/S checkpoint requires membrane hyperpolarization, which is accomplished by the outflow of K+ [295]. Together with its regulatory subunit, KCNE3, the potassium channel KCNQ1 plays a crucial role. In order to recycle potassium at the basolateral membrane, which is necessary for membrane repolarization, KCNQ1/KCNE3 voltage-gated channels are required [296]. KCNQ1 functions as a tumor suppressor gene that controls cellular growth, innate immunity, signaling pathways that control growth, and inflammation.

When lost or inactive, KCNQ1 acts as a tumor suppressor, because it prevents the development of cancer [297]. In addition to membrane hyperpolarization, KCNQ1 is also involved in inflammatory response, oxidative stress, stem cell homeostasis, growth regulation signaling pathways, and ion channel function, all of which are associated with oncogenesis. The downregulation of lipid oxidation caused by KCNQ1 knockout has profound impacts on lipid metabolism [298], and the transition to lipogenesis as a result of the suppression of fatty acid oxidation appears to be closely linked to oncogenesis.

In KCNQ1 KO and MUC2 KO mice, several oxidative stress-related genes, including cytochrome oxidase P450 enzymes and various glutathione transferases, appear to be dysregulated [298]. In colon epithelial cells, estrogen encourages the phosphorylation of KCNQ1 and the sequestration of the channel into endocytic vesicles [299]. The redistributive process only affects females and has no impact on the overall abundance of KCNQ1. In CRC, KCNQ1 is gradually becoming recognized as a key tumor suppressor. Better CRC survival is related to sustained KCNQ1 expression, and KCNQ1 overexpression reduces nuclear catenin accumulation [300,301].

Finally, the regulatory sulfonylureas receptor (Sur1, Sur2, and their splicing products Sur2A and 2B) subunits are coupled with the inwardly rectifying K+ subunits Kir6.1 and Kir6.2 and form the ATP-sensitive potassium (KATP) channel complexes. Low intracellular ATP/ADP ratios, second messengers, kinases such as AMPK, and hormones, are all known to trigger KATP channels [302]. Estrogens may regulate Kir6.2 and Sur2A-B differently in some tissues; for example, they may upregulate them in cardiomyocytes and decrease KATP channel subunits in neurons, as has been shown in female rats with different effects [303]. The KATP channel subunits are functionally expressed in a variety of cancer cell types, including hepatocellular carcinoma [304], human bladder cancer, human gastric cancer, and glioma [305]. This has been demonstrated by in vitro and ex vivo research. In two animal models of cancer, the Sur2A component was expressed more highly in proliferating cells.

The Kir6.1/2-Sur2A/B subunits are a therapeutic target in breast and kidney malignancies, according to immunohistochemistry/omics/pharmacovigilance data [306].

In summary, our survey highlights that greater knowledge of the molecular mechanisms underlying the gender-related disparities in cancer would likely lead to higher levels of precision medicine and improved treatment options for both males and females with neoplastic disorders.

## Figures and Tables

**Table 1:** Differences in oxidative stress in females.

Study Type	Subject of the Investigation	Variations of Oxidative Stress in Females	Effects	Ref.
In vivo animal model	*Drosophila melanogaster*	Reduced ROS production and increased antioxidant enzymes	Longer lifetime	[21]
	Mice and rats	Reduced mitochondria release of superoxide radicals	Longer lifetime	[24,26,27,28]
	Rats	Reduced NADPH oxidase activation. Lower levels of p47	Effects on circulation	[29]

**Table 2:** Different effects of experimental models of liver damage in the two sexes.

Experimental Model	Animals	Effects on Males	Results	Ref.
Diethylnitrosamine and ethanol	Mice	Decreased antioxidant capacity	Increased incidence of liver cancer	[122]
Aminobiphenyl	Mice	Reduced antioxidant gene expression and increased oxidative damage. Altered nuclear factor erythroid-2-related factor 2. Reduced defense against carcinogen-induced liver carcinogenesis. Reduced immune response to infections. Altered expression of inflammatory cytokines.	Increased incidence of liver cancer	[128,129,130,131]
Aflatoxin B1 (AFB1)	Glutathione S-transferase A3 Knockout mice	Reduced AFB1-DNA adducts	Reduced vulnerability to liver cancer development.	[136]

**Table 3:** Effects of different experimental models of lung damage.

Experimental Model	Subjects of Analysis	Effect	Results in Females	Ref.
Naphthalene	Mice	Increased ROS production. Increased CYP enzyme expression	More airway damage	[197]
Polycyclic aromatic hydrocarbons (PHAs)	Mice	Oxidative damage to DNA. PHA-DNA adducts formation.	Increased oxidative stress	[200,201,202]
	Humans	Women are more vulnerable than males to oxidative stress and chromosomal damage by PHAs.	Increased lung cancerogenesis	[203]

**Table 4:** Different risk rates of cancer according to oxidative stress mechanisms in males and females.

	Higher Risk	Mechanism	References
Glioma	men	Testosterone has neurotoxic effects	[107]
Liver cancer	men	Low levels of alcohol dehydrogenase	[121]
Colorectal cancer	women	Low levels of unconjugated bilirubin, which has antioxidant effects	[173]
Lung cancer	smokimg women	High levels of CYP1A1 and CYP1B1 that activate tobacco smoke components to create ROS	[194]
Melanoma	men	Low levels of antioxidant enzymes in the skin	[225]
Non-Hodgkin Lymphoma	men	High levels of superoxide in thymocytes overexpressing Bax	[233]

## Data Availability

Not applicable.
